# Desmoid tumour of the rectus sheath following myomectomy: a mimicker of parasitic leiomyoma

**DOI:** 10.1093/jscr/rjag655

**Published:** 2026-07-29

**Authors:** Patrick D Melmer, Akhila Kunuthuru, Gati Wambura

**Affiliations:** Department of Surgery, Virginia Commonwealth University, Richmond, VA, United States; Virginia Commonwealth University, Richmond, VA, United States; Virginia Commonwealth University, Richmond, VA, United States

**Keywords:** desmoid, parasitic leiomyoma, myomectomy, rectus sheath, case report

## Abstract

Desmoid-type fibromatosis is a rare, locally aggressive soft tissue tumour that may mimic other abdominal wall lesions. Distinguishing desmoid tumours from parasitic leiomyomas can be challenging, particularly in women with prior gynecologic surgery. A 34-year-old woman with a history of Pfannenstiel myomectomy presented with a progressively enlarging, painful left lower quadrant abdominal wall mass. Magnetic resonance imaging demonstrated a 10.5 × 8.3 × 9.4 cm lesion within the left rectus sheath concerning for recurrent leiomyoma. Given persistent symptoms and concern for parasitic leiomyoma, she underwent surgical excision with primary fascial repair. Histopathology revealed desmoid-type fibromatosis, characterized by nuclear β-catenin positivity and absence of smooth muscle marker expression. Abdominal wall desmoid tumours may closely resemble parasitic leiomyomas clinically and radiographically. Definitive diagnosis requires histopathologic and immunohistochemical evaluation. Management should be individualized and supported by multidisciplinary surveillance due to the risk of local recurrence.

## Introduction

Desmoid tumours, or desmoid-type fibromatoses, are rare fibroblastic proliferations arising from musculoaponeurotic structures [1, 2]. Although histologically benign, they are locally aggressive and prone to recurrence [1–3]. Sporadic cases often harbor CTNNB1 mutations, resulting in aberrant nuclear β-catenin accumulation, while familial cases occur in familial adenomatous polyposis [1, 2]. Abdominal wall desmoids frequently arise in women of childbearing age, often in relation to prior surgery or pregnancy [1, 2].

Parasitic leiomyomas are extrauterine smooth muscle tumours that can detach from the uterus and implant on peritoneal or abdominal wall surfaces [4]. Clinically and radiographically, they may mimic desmoid tumours [4]. Magnetic resonance imaging (MRI) can aid localization and assessment of extent but is not definitive; histopathologic evaluation with immunohistochemistry is often required [4].

Recent literature emphasizes active surveillance for asymptomatic or stable desmoid tumours [2, 5]. Surgery is indicated for symptomatic or progressive lesions, ideally achieving complete (R0) resection, while balancing functional outcomes and recurrence risk, as in our case described below [1–3, 5]. Multidisciplinary management is essential [1, 2]. This case report has been reported in line with the Surgical CAse REport (SCARE) checklist [6].

## Presentation of case

**Figure 1:**
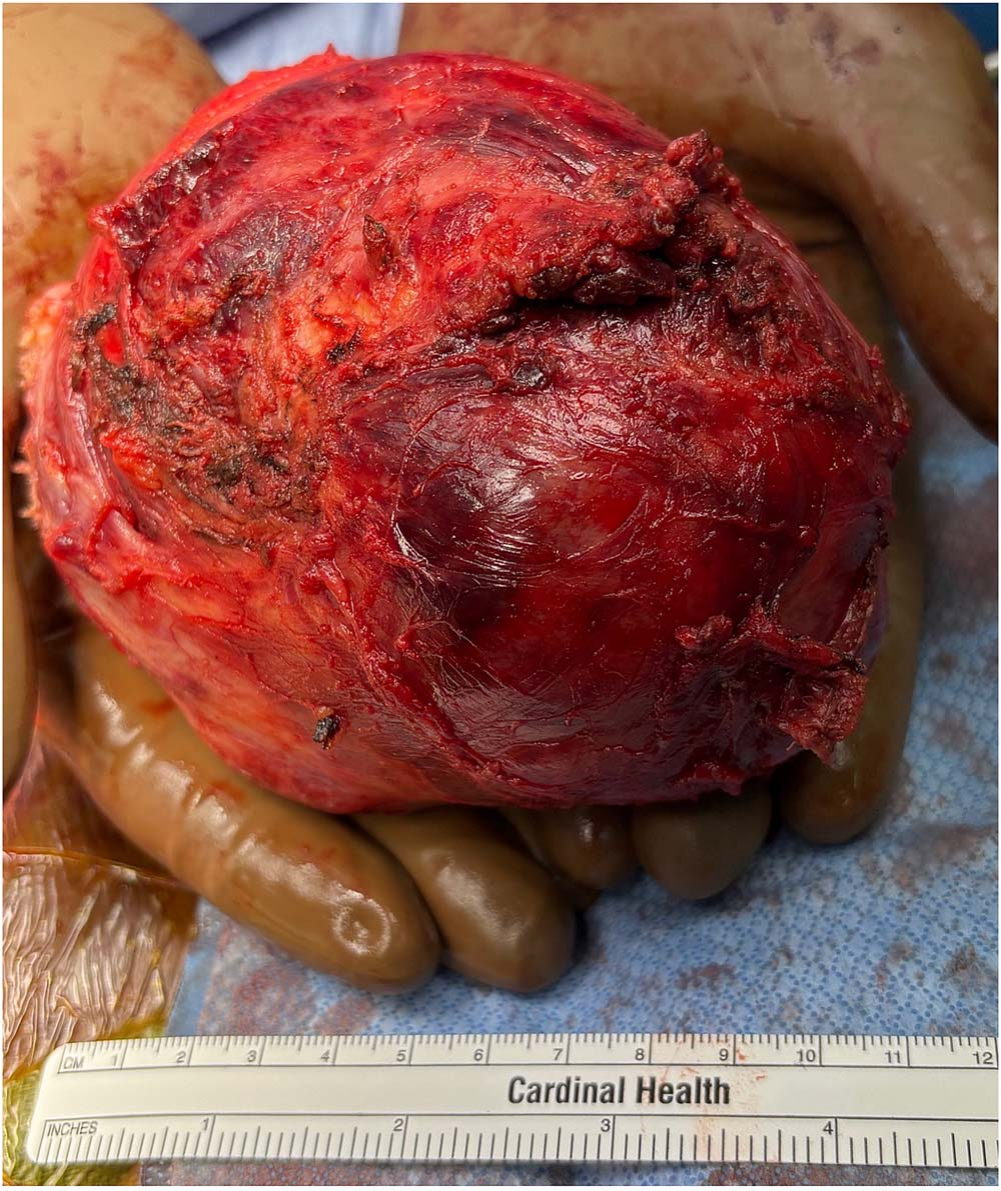
Intraoperative photo of resected mass.

A 34-year-old woman presented with a progressively enlarging, painful left lower quadrant abdominal wall mass. She had undergone Pfannenstiel myomectomy in Kenya 2 years prior, with an uncomplicated recovery. No systemic symptoms were reported. Pelvic MRI demonstrated a 10.5 × 8.3 × 9.4 cm well-circumscribed, enhancing soft-tissue mass within the left rectus sheath, displacing the rectus muscle laterally. The lesion exhibited intermediate T2 signal with mild heterogeneity and no necrosis or diffusion restriction. Differential diagnosis included parasitic leiomyoma versus desmoid-type fibromatosis. Patient-centered multidisciplinary discussion was held with the decision made to proceed with operative intervention given progressive and recurrent symptoms. In the OR, the mass was firm, well-circumscribed, and adherent to the posterior rectus sheath. Circumferential dissection allowed en bloc resection, with partial posterior sheath excision (Fig. 1). The fascial defect was repaired primarily, closed-suction drains were placed, and the procedure overall tolerated well. The patient recovered uneventfully and was discharged on postoperative Day 2. Drains were removed sequentially. Gross examination revealed a 12 cm encapsulated mass. Histology demonstrated uniform spindle cells in fascicles with abundant collagen, without atypia or necrosis. Immunohistochemistry was nuclear β-catenin positive and desmin/smooth-muscle actin negative, confirming desmoid-type fibromatosis. At 3-month follow-up, she had complete resolution of pain and no evidence of recurrence. She was referred to surgical oncology for continued surveillance.

## Discussion

Abdominal wall desmoids may mimic parasitic leiomyomas [4]. Imaging is helpful for localization and surgical planning but is insufficient for definitive diagnosis [4]. MRI features including size, signal characteristics, and enhancement patterns can suggest a diagnosis but cannot distinguish leiomyoma from desmoid [4]. Histopathology with immunohistochemistry remains the gold standard: desmoids exhibit nuclear β-catenin positivity, whereas leiomyomas stain for smooth-muscle markers [1, 2, 4].

Abdominal wall desmoids predominantly affect women of reproductive age and are often associated with prior surgery or trauma [1, 2]. Parasitic leiomyomas share similar epidemiologic associations, highlighting the need for a broad differential in patients with prior uterine procedures [4]. Here the history of prior abdominal wall leiomyoma excision created a unique situation where local trauma and prior surgery likely added to the development of desmoid tissue. While current paradigms include active surveillance for asymptomatic or stable tumours, the recurrent nature of our case necessitated operative resection [2, 5]. Systemic options including nonsteroidal anti-inflammatory drugs, anti-estrogen agents, tyrosine kinase inhibitors, and chemotherapy are reserved for unresectable or multiply recurrent lesions [1, 3, 5]. Multidisciplinary care is essential for treatment planning [1, 2].

## Conclusion

Desmoid-type fibromatosis of the abdominal wall is rare and may mimic parasitic leiomyomas. This case highlights the diagnostic and therapeutic complexities of abdominal wall desmoid tumours, particularly in patients with prior gynecologic surgery. Multidisciplinary evaluation, individualized treatment, and structured long-term surveillance optimize outcomes.
